# Denoising of neuronal signal from mixed systemic low-frequency oscillation using peripheral measurement as noise regressor in near-infrared imaging

**DOI:** 10.1117/1.NPh.6.1.015001

**Published:** 2019-01-09

**Authors:** Stephanie Sutoko, Yee Ling Chan, Akiko Obata, Hiroki Sato, Atsushi Maki, Takashi Numata, Tsukasa Funane, Hirokazu Atsumori, Masashi Kiguchi, Tong Boon Tang, Yingwei Li, Blaise deB Frederick, Yunjie Tong

**Affiliations:** a Hitachi, Ltd., Center for Exploratory Research, Research & Development Group, Akanuma, Hatoyama, Saitama, Japan; b Universiti Teknologi PETRONAS, Electrical and Electronic Engineering Department, Bandar Seri Iskandar, Tronoh Perak, Malaysia; c McLean Hospital, Brain Imaging Center, Belmont, Massachusetts, United States; d Yanshan University, School of Information Science and Engineering, Qinhuangdao, China; e Harvard Medical School, Department of Psychiatry, Boston, Massachusetts, United States; f Purdue University, Weldon School of Biomedical Engineering, West Lafayette, Indiana, United States

**Keywords:** low-frequency oscillation, brain, peripheral, near-infrared spectroscopy, systemic noise, denoising, working memory

## Abstract

Functional near-infrared spectroscopy (fNIRS) is a noninvasive functional imaging technique measuring hemodynamic changes including oxygenated ($O_{2}\mathrm{Hb}$) and deoxygenated (HHb) hemoglobin. Low frequency (LF; 0.01 to 0.15 Hz) band is commonly analyzed in fNIRS to represent neuronal activation. However, systemic physiological artifacts (i.e., nonneuronal) likely occur also in overlapping frequency bands. We measured peripheral photoplethysmogram (PPG) signal concurrently with fNIRS (at prefrontal region) to extract the low-frequency oscillations (LFOs) as systemic noise regressors. We investigated three main points in this study: (1) the relationship between prefrontal fNIRS and peripheral PPG signals; (2) the denoising potential using these peripheral LFOs, and (3) the innovative ways to avoid the false-positive result in fNIRS studies. We employed spatial working memory (WM) and control tasks (e.g., resting state) to illustrate these points. Our results showed: (1) correlation between signals from prefrontal fNIRS and peripheral PPG is region-dependent. The high correlation with peripheral ear signal (i.e., $O_{2}\mathrm{Hb}$) occurred mainly in frontopolar regions in both spatial WM and control tasks. This may indicate the finding of task-dependent effect even in peripheral signals. We also found that the PPG recording at the ear has a high correlation with prefrontal fNIRS signal than the finger signals. (2) The systemic noise was reduced by 25% to 34% on average across regions, with a maximum of 39% to 58% in the highly correlated frontopolar region, by using these peripheral LFOs as noise regressors. (3) By performing the control tasks, we confirmed that the statistically significant activation was observed in the spatial WM task, not in the controls. This suggested that systemic (and any other) noises unlikely violated the major statistical inference. (4) Lastly, by denoising using the task-related signals, the significant activation of region-of-interest was still observed suggesting the manifest task-evoked response in the spatial WM task.

## 1 Introduction

Functional near-infrared spectroscopy (fNIRS) is an imaging technique that noninvasively measures the product of cerebral hemodynamics (concentration changes of oxygenated and deoxygenated hemoglobin; $\Delta C_{O_{2}\mathrm{Hb}}$ and $\Delta C_{\mathrm{HHb}}$) and optical path length (L), using light in the near-infrared spectrum (650 to 900 nm).^1^^–^^3^ Because hemodynamic changes are related to local neuronal activity through neurovascular coupling,^4^^,^^5^ fNIRS is commonly used to interpret brain activity and function. fNIRS has been widely used in research, clinical, and educational purposes^6^ due to its cost-effectiveness, safety, flexibility, higher spatial resolution than electroencephalography, and better temporal resolution than functional magnetic resonance imaging (fMRI).^7^ fNIRS systems are more compact than fMRI, which enables practical and continuous bedside monitoring even in infants and young children.^8^^–^^10^ fNIRS provides better motion tolerance; therefore, fNIRS is suitable for examining the challenging patients with restless symptoms^11^^,^^12^ and subjects actively engaging in movement such as walking and running.^13^^,^^14^ Furthermore, cochlear implant patients can safely undergo fNIRS measurement because there is no magnetic field that may endanger patients.^15^^,^^16^

Despite those advantages, there are three confounding factors in fNIRS studies. These factors are: (1) the mixture of neuronal and systemic physiological (nonneuronal) signals which is found in the low frequency (LF) range^17^^,^^18^ especially when the activation period is relatively short.^19^^,^^20^ This nonneuronal signal is compounded by several sources such as Mayer waves and vasomotion.^21^^–^^25^ In addition, because fNIRS data are measured through the intact skull, so every fNIRS channel has extracerebral noises (from skin, skull, and blood vessels on the surface of the brain). As a result, fNIRS is more susceptible to systemic noises. (2) Performing tasks likely incorporates not only targeted activation but also untargeted ones in the nearby regions.^26^^,^^27^ Since fNIRS has a low spatial resolution ($>{\mathrm{cm}}^{3}$) with limited channels, and each channel covers a large area (i.e., low spatial resolution), it is prone to detect both targeted and untargeted activation within the same channel. (3) The mixture neuronal and systemic physiological signals in the LF band, coupled with small sample numbers may affect statistical inferences.^28^ As a recent study pointed out,^29^ false-positive rates in many neuroimaging studies were largely inflated. Moreover, Hocke et al.^30^ demonstrated that some data processes, such as low-pass filtering, can also artificially inflate statistical power. In summary, obtaining accurate brain activation from fNIRS requires (1) careful experimental design with one or more built-in controls and (2) effective denoising methodologies used in both data acquisition and analyses.

Regarding the problem of an equivocal signal mixture, many efforts have been made to denoise fNIRS signal; we can classify these efforts into four approaches. First, the intracranial signal is particularly extracted by regressing the extracerebral effects using multidistance (MD) short-detector (S-D) separations.^31^^,^^32^ This approach measures blood-related changes in the superficial layer (e.g., scalp and skull) by using the S-D separation and successfully eliminates extracerebral noises;^33^^,^^34^ however, systemic noise still remains, as it is present not only in the superficial but also in the deep (i.e., cortices) layers.^35^^,^^36^ Second, many studies attempted the advanced computational analysis to isolate the systemic noises from brain signals. Yamada et al.^37^ proposed the separation between functional and systemic signals based on the assumption of the negative and positive relationship between $O_{2}\mathrm{Hb}$ and HHb, respectively. Prince et al.^38^ applied a model of systemic and cerebral activity components with the assumption of exact frequencies in the state space estimation techniques. This method is limited by noise-stimulus phase-locking,^17^^,^^18^ and simple component modeling likely underestimate the signal complexity. Third, data-driven methods such as principal and independent component analyses (PCA and ICA) have been introduced to decompose mixed signals into subsets of statistically uncorrelated and independent components (ICs), respectively.^39^^–^^41^ The empirical component^42^^,^^43^ can be selected by maximizing the correlation and covariance of ICs in the repeated stimulus frame.^44^ However, the problem of noise-stimulus phase-locking again influenced ICs selection and signal reconstruction. Therefore, the assumption of statistical independence among ICs could be biased against the characteristics of systemic noise in the event of brain activation. Finally, systemic physiological signals (e.g., respiration rate and arterial blood pressure) are measured using multimodalities to provide accurate noise regressors.^45^^,^^46^ However, the systemic noise regressor is restricted by the number of physiological signal recordings.^25^ In addition, the excessive multimodal measurement might burden subject’s convenience.

In this study, we explored several ways to improve the accuracy of fNIRS experiments. We used the spatial working memory (WM) task as one of the cognitive measures. For experimental design, first, we incorporated the systematic control task (i.e., motor control) to avoid untargeted brain activation, which can arise from a synchronized movement associated with the spatial WM task. Second, we also incorporated the resting state task as a control to rule out false positives. Third, we developed a denoising method based on a general linear model (GLM)^47^^,^^48^ using the simultaneous peripheral photoplethysmographic (PPG) measurement (e.g., ear and finger) as systemic noise regressors. By directly recording the peripheral signals that we believe contained only the systemic noise, we avoided the error of noise modeling and do not require the questionable assumption of independent noise components. We chose the ear and finger to be the noise regressors for the following reasons. (1) Based on previous concurrent fMRI/fNIRS research,^49^ the $O_{2}\mathrm{Hb}$ signal recorded from finger has broad and high correlations with blood oxygen level dependent fMRI signals in the brain. (2) We believe that, compared to the finger, the ear, which is much closer to the brain on the vascular path, should share more systemic fluctuations with the brain. (3) There are no confounding neuronal signals in these peripheral sites as mentioned above. (4) These locations are easy to measure by peripheral PPG device.

## 2 Materials and Methods

### 2.1 Subjects

Seventeen healthy adults (6 females, 11 males, age=40.1±11.1 y.o.; mean±SD, range=24 to 57 y.o.) participated in this study. Their handedness was assessed with the Edinburgh Handedness Inventory:^50^ 16 subjects were right-handed. Data were obtained according to the standards of the internal review board of the Research and Development group, Hitachi, Ltd. All subjects received a detailed explanation of the measurements to be performed and provided the informed consent before participating in this experiment. Unexpected technical problems occurred, and the affected data (one and two samples for spatial WM and motor control tasks, respectively) were excluded in data analysis.

### 2.2 Task Paradigms

**Fig. 1:**
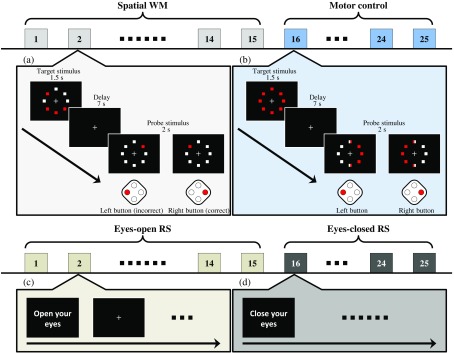
Two measurement sessions (spatial WM, motor control; eyes-open, eyes-closed RS). Each session consisted of 25 trials; 15 trials for spatial WM/eyes-open RS and 10 trials for motor control/eyes-closed RS. (a) In the spatial WM task, subjects responded the probe stimulus by matching the position of red square in the target stimulus. (b) Meanwhile, the target stimulus was not required to be encoded and retrieved because subjects were merely required to answer which side majorly showed white squares during motor control. In the RS, subjects rested while (c) kept their eyes opened and (d) then closed their eyes.

Each subject was measured in two sessions as shown in Fig. 1. Each session lasted for 15 min. All tasks were designed using the Platform of Stimuli and Tasks software (Hitachi Ltd., Central Research Lab.) and presented on a monitor put in front of subjects. The first session involved 15 trials of spatial WM task and 10 trials of motor control task [Figs. 1(a) and 1(b)]. As in previous spatial WM studies,^51^ subjects were asked to encode the position of four red squares among displayed eight squares within 1.5 s (target stimulus) and to maintain the spatial information for 7 s. During the memory maintenance period, subjects kept their sight on a fixation cross appearing on the black background screen. The monitor displayed a red square among eight squares (the probe stimulus) after the maintenance period. In addition to evoking WM, subjects were required to switch into decision-making mode to retrieve information and respond.^52^^,^^53^ Subjects needed to indicate whether the position of the red square in the probe stimulus was identical with the target stimulus [Fig. 1(a)] within 2 s. Since subjects gave responses by pressing buttons, we were aware of motor-evoked activation in the motor cortex.^2^^,^^54^ In order to estimate the impact of button pressing (motor) alone, a motor control task (with no information encoding) was given following the spatial WM task (still in session 1). The motor control was designed to always show all the red squares in the target stimulus and a fixation cross during the maintenance period. In the probe stimulus, we showed white squares on the left and red on the right (or vice versa) and asked the subjects to press button according to the location of the white squares [e.g., press left button if the white squares were on the left, see Fig. 1(b)]. The intertrial interval was randomized from 16 to 24 s. In the second session, the resting state without any target and probe stimulus was used as another control task [Figs. 1(c) and 1(d)]. While resting, subjects fixed their sight on a fixation cross for the first 8 to 9 min [similar to the period for 15 trials; Fig. 1(c)] and closed their eyes for the remaining time [Fig. 1(d)]. The end of the second session was marked by soft beeping.

### 2.3 Measurements

**Fig. 2:**
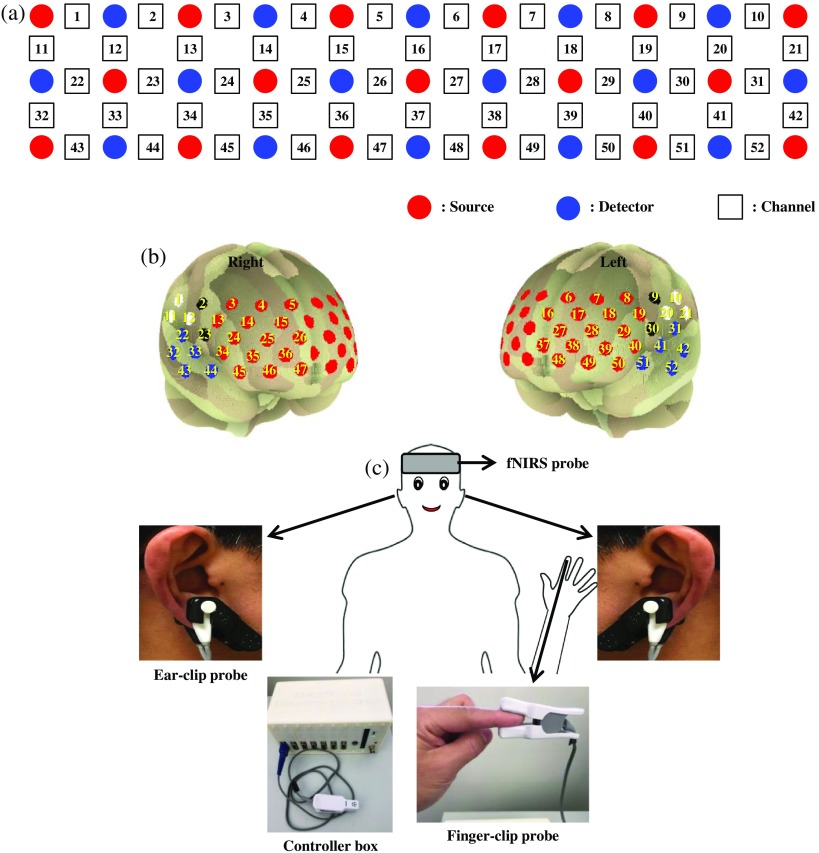
(a) A plane of fNIRS probe holder with 3×11 configuration of source-detector (b) measured 52 channels that were spatially registered in frontal cortex. The frontal cortex was classified into four major channel regions: bilateral postcentral (i.e., white-colored channels), bilateral premotor (black-colored channels), bilateral temporal (blue-colored channels), and frontopolar (red-colored channels). The fNIRS probe was put on the subject’s forehead whereas the PPG with controller box was connected to ear- and finger-clip probes fixed in subject’s ears and left index finger.

Prefrontal measurements were acquired using an ETG-4000, a dual wavelength (695 and 830 nm) fNIRS system (Hitachi Medical Corporation, Tokyo, Japan), with a sampling rate of 10 Hz. One plane probe [Fig. 2(a), 3×11 S-D arrangement; 17 emitters and 16 detectors] with a 52-channel system was put on the subject’s frontal lobe. The coordinates of the measured regions (i.e., channels) were estimated in the middle of each S-D pair and illustrated by the template of spatial registration [Fig. 2(b)].^55^^,^^56^ Channels were categorized into four major regions based on the macroanatomy classification: bilateral postcentral, bilateral premotor, bilateral temporal, and frontopolar. The peripheral measurement was simultaneously conducted using multichannel PPG device (McLean Hospital, Massachusetts). This device was equipped with Nellcor type D-YS (ear-clip sensor) and DS-100A pulse oximeter (finger-clip sensor) probes with dual wavelengths (660 and 920 nm) and sampling rate 31.25 Hz.^57^ Ear-clip probes were attached to both ears and a finger-clip sensor was put on the left index finger [Fig. 2(c)].

### 2.4 Data Preprocessing and Analysis

**Fig. 3:**
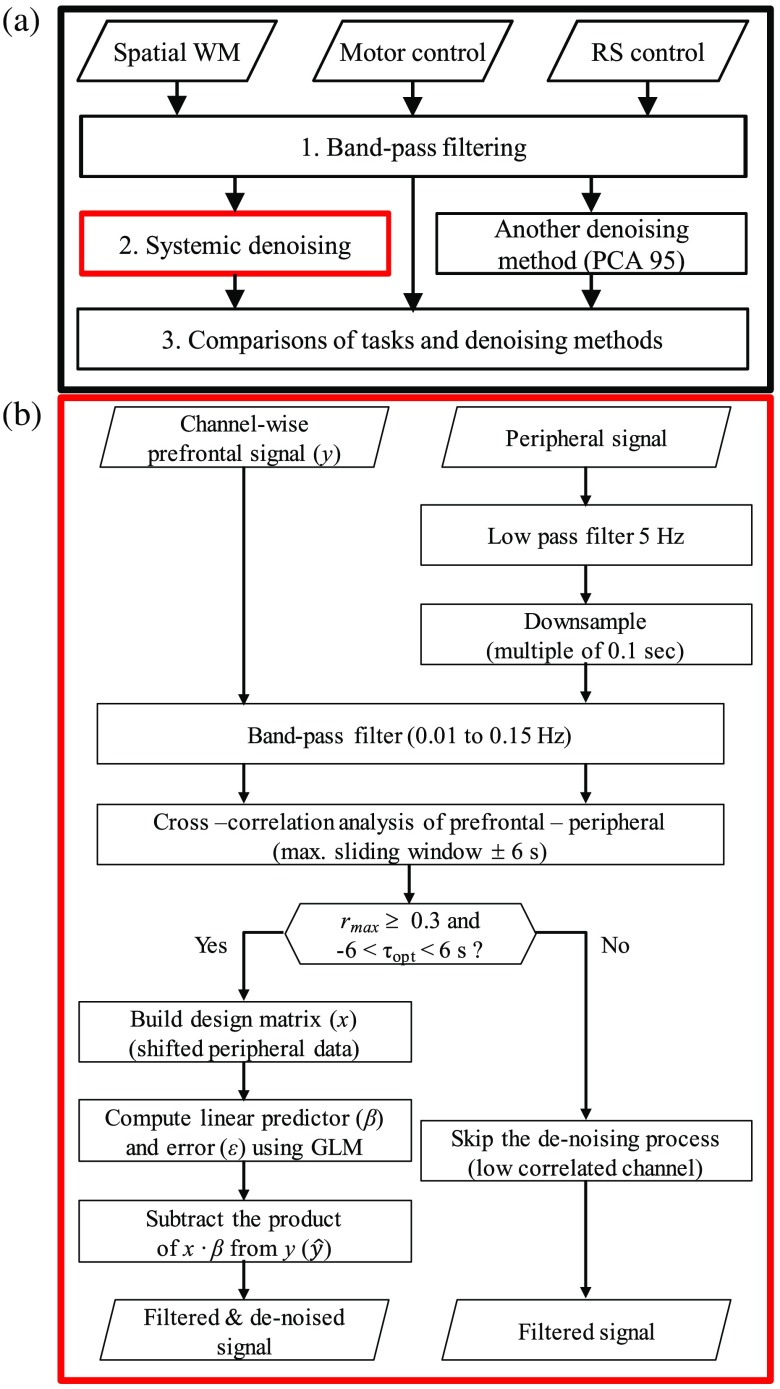
(a) Overall summary of data analysis and (b) detail flow-chart for GLM-based systemic denoising process.

Analyses were computed using MATLAB (The MathWorks, Inc.) and plug-in-based analysis platform, Platform for Optical Topography Analysis Tools (POTATo, developed by Hitachi Central Research Laboratory).^58^ Measured data from prefrontal and peripheral sites were both initially converted to the product of hemoglobin concentration changes and optical path length (ΔC·L) for three signal types $O_{2}\mathrm{Hb}$, HHb, and Hb-total following the modified Beer–Lambert equation. ^2^^,^^59^ Figure 3(a) shows the flowchart of the analysis summary in which three analysis steps were performed.

#### 2.4.1 Processing without systemic denoising

**Fig. 4:**
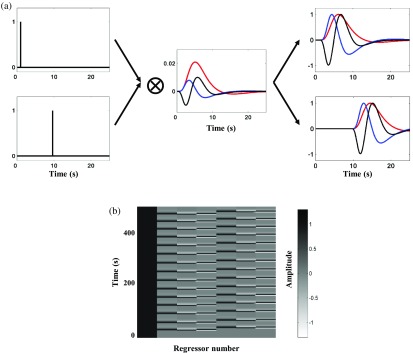
(a) Construction of neuronal activation regressors based on HRF (red plot for canonical HRF) and its derivatives (temporal and dispersion derivatives; blue and black plots, respectively) convolved with two event-designs of memory encoding and button pressing. (b) An example of regressor matrix design (x) with orders of constant, encoding canonical HRF, encoding temporal, encoding dispersion, button-pressing canonical, button-pressing temporal, and button-pressing dispersion derivatives.

The continuous and channelwise fNIRS signal was bandpass filtered (0.01 to 0.15 Hz; zero-phase Butterworth).^51^ The activation was estimated on the basis of the hemodynamic response function (HRF) in GLM analysis

y=xβ+ϵ (1)

where $y\in R^{L\times ch}$ (L = length of time points, ch = channel numbers) is fNIRS signals. $x\in R^{L\times N}$ is the regressor matrix (N = number of regressor) designed by a constant, convolution between the boxcar function and HRF functions (i.e., canonical two-gamma, temporal and dispersion derivatives). The boxcar function corresponds to the time-piece of events (i.e., memory encoding and button pressing) as shown in Fig. 4(a). $\beta \in R^{N\times ch}$ is the estimated linear regressor of x by the least square error, normally distributed $\epsilon \in R^{L\times ch}$ with 0 mean and $\sigma^{2}$ variance:^60^

$$h(t)=\frac{t^{\tau_{p}}e^{-t}}{(\tau_{p})!}-\frac{t^{\left(\tau_{p}+\tau_{d}\right)}e^{-t}}{A(\tau_{p}+\tau_{d})!},$$ (2)

where h is the time (t) function of canonical two-gamma HRF,^61^ $\tau_{p}$ is the parameter of first peak delay, $\tau_{d}$ is the parameter of second undershoot peak delay, and A is the amplitude ratio between the first and second peaks. We used the typical parameters of 6, 10, and 6 for $\tau_{p}$, $\tau_{d}$, and A, respectively. Figure 4(b) shows the example of a regressor matrix. The model significance was statistically evaluated as follows:

Tstat=c′βσ^2c′(x′x)−1c, (3)

σ^2=(y−xβ)′(y−xβ)L−p, (4)

where $T_{\text{stat}}$ is the t-statistic value, c is the contrast vector to infer the effect of canonical two-gamma HRF (i.e., c = [0 1 0 0 0 0 0]), σ^2 is the sum of squared error values corrected by L−p degree of freedom (p = rank x).^62^^–^^64^ The channel significance was statistically evaluated in random effect group analyses using a one-sample t-test of estimated β-values for the HRF regressor against zero. The strength of the phenomenon was assessed using the parameter of Cohen’s d effect size. The subject-level analysis evaluated the HRF $T_{\text{stat}}$ with L−p degree of freedom.

#### 2.4.2 Processing with systemic denoising

Systemic denoising was done for each task [Fig. 3(b)]. Therefore, after matching the sampling rate between fNIRS and peripheral PPG recordings, the continuous data were cut after the first 15 trials data, band-pass filtered in the LF window (0.01 to 0.15 Hz; zero-phase Butterworth), and linearly detrended. The relationship between (channelwise) prefrontal fNIRS and peripheral PPG signals (i.e., $O_{2}\mathrm{Hb}$ and HHb) was assessed using the cross-correlation analysis. The maximum correlation ($r_{\text{max}}$) was determined within the confined sliding window ±6  s based on the physiological characteristic of vascularization.^49^ The poor-quality signal may cause spurious correlations. According to a previous study,^30^ filtering and optimum delay search (i.e., cross correlation) elevate the correlation coefficient. In order to minimize the risk of spurious correlation, the threshold of $r_{\text{max}}$ was set to be 0.3 (p<0.01). The channelwise prefrontal signal having $r_{\text{max}}$ less than 0.3 or greater than 0.3 with optimum delay ($\tau_{\mathrm{opt}}$) at the boundary of sliding window (either −6 or 6 s) would left untouched. For denoising case, the peripheral signals would be shifted according to the delay (corresponding to the $r_{\text{max}}$) to construct the systemic noise regressors. The use of dual peripheral regressors was also evaluated in which ear and finger signals were shifted independently. The systemic noise regressors were then combined with the typical x regressor matrix (Fig. 4). Activation analysis was conducted following step 1 as β-values for the HRF regressor was also obtained while performing denoising. The signal reduction caused by the denoising process was evaluated to examine the portion of systemic noise in fNIRS signal by the following equations:

SNP|ch=1Δt∫(yt,ch−y^t,ch)2dt1Δt∫(yt,ch)2dt, (5)

y^=y−x(Iβ) (6)

where SNP is the systemic noise portion, which is defined as the ratio between the square root of average signal difference caused by denoising (y−y^) and the raw signal y in all time-point t for each channel ch, y^ is the denoised signal, and $I\in R^{N\times N}$ is a square matrix in which diagonal elements associated with systemic noise and constant regressors are one. The efficiency of denoising was assessed through the change of activation significance and spatial distribution.

#### 2.4.3 Comparisons of tasks and denoising methods

Both spatial WM and control (i.e., motor and resting state) tasks were analyzed in the same way as introduced in processing Sec. 2.4.1 without and Sec. 2.4.2 with systemic denoising. In the motor control task, the result would interpret the activation pattern caused by button pressing, since no WM was involved. In the resting state task, the block design was invisible to subjects (only known by the computer). In our limited sample number, this resting state with no significant activation would assess the risk of the false-positive result. The comparison among tasks (e.g., peripheral correlation and signal reduction) was statistically done using the analysis of variance (ANOVA). In order to evaluate the current denoising feasibility, these datasets were also preprocessed by an existing method, PCA.^43^ PCA was performed independently for each signal type (i.e., $O_{2}\mathrm{Hb}$ and HHb). For component selection, PCA neglected components with total contribution less than 5%.

## 3 Results

### 3.1 Brain Activation in the Processing without Systemic Denoising

**Fig. 5:**
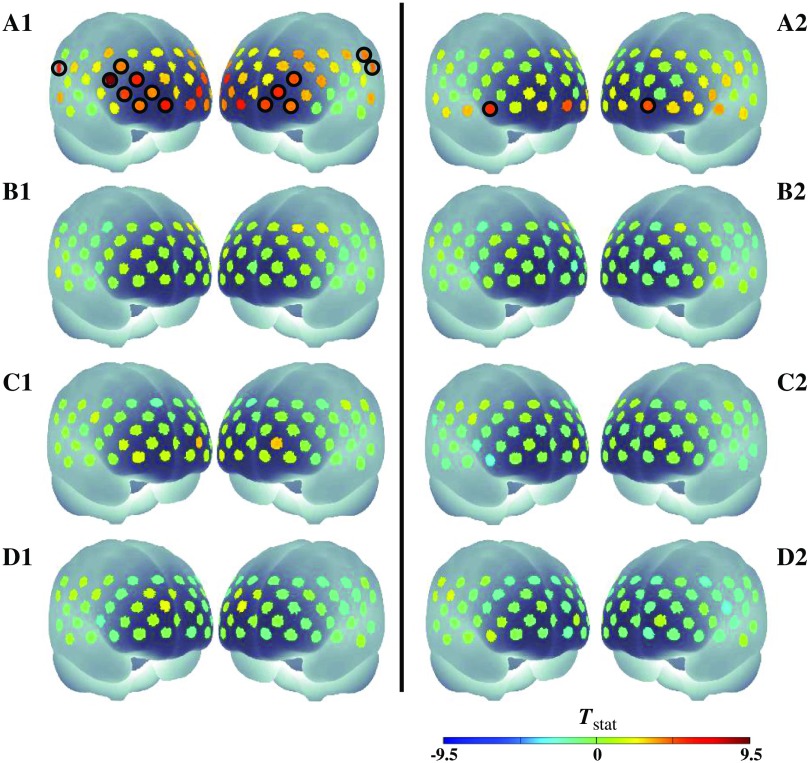
$T_{\text{stat}}$ maps of $O_{2}\mathrm{Hb}$ (A1–D1) and HHb (A2–D2) activations for spatial WM (A1–2), motor control (B1–2), eyes-open (C1–2), and eyes-closed RS (D1–2) before denoising. Black encircled channels indicate the channels with significant activation (p<0.05; Holm–Bonferroni correction) in one-sample t-test.

We found the significantly increased $O_{2}\mathrm{Hb}$ activations (p<0.05; Holm–Bonferroni corrected) only in the spatial WM task but not in any control tasks [Figs. 5(a)–5(d)]. For the spatial WM task, the increase of $O_{2}\mathrm{Hb}$ activation was significantly seen in both middle frontal (Broadmann area/BA 10, 45, 46, and 11) and bilateral temporal supramarginal areas, with the strongest activation observed in the right ventrolateral prefrontal cortex (VLPFC, channel 24, right BA 45, $T_{\text{stat}}=9.37$). HHb activation was also investigated in group analysis; however, there was no significant decrease observed in any tasks. Oppositely, we found minor significant HHb increases in the lower frontopolar only in the spatial WM task. We confirmed the significance of individual data. All subjects (i.e., 16) showed the significantly increased $O_{2}\mathrm{Hb}$ activation (p<0.05) in right BA 45 (i.e., channels 24). Seven out of 16 subjects showed the significantly decreased HHb activation (p<0.05) in right BA 45 as performing the spatial WM task. From these results, we classified three response types toward the spatial WM task in BA 45: (1) seven subjects having both significant $O_{2}\mathrm{Hb}$ increase and HHb decrease, (2) eight subjects having significant $O_{2}\mathrm{Hb}$ and HHb increases, and (3) a subject having significant $O_{2}\mathrm{Hb}$ increase and null HHb decrease. This suggested that the $O_{2}\mathrm{Hb}$ response was more uniform compared to the HHb response during the spatial WM task. Despite the various hemodynamic responses in the spatial WM task, neither significant $O_{2}\mathrm{Hb}$ increase nor HHb decrease was found during control tasks. The finding that the spatial WM task alone showing significant activation suggested that (1) the presence of spontaneous LF systemic noise (and any other noises) does not evoke false-positive results in the group-analysis of control tasks and (2) fNIRS signal during the spatial WM task does reflect encoding-evoked response, neither the sham encoding (i.e., motor control task) nor the motor-related activation (i.e., button-pressing).

### 3.2 Temporal Correlation between Prefrontal and Peripheral Signals

**Fig. 6:**
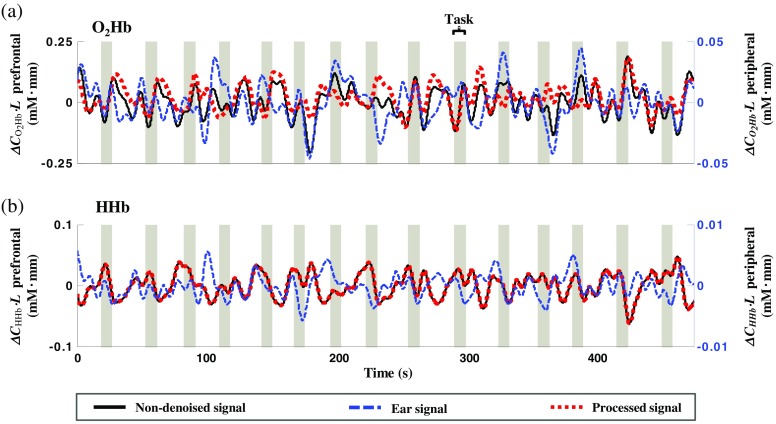
Representative temporal traces of (a) $O_{2}\mathrm{Hb}$ and (b) HHb prefrontal (solid black line, left y-axis scale) and peripheral ear (blue dashed-line, right y-axis scale) signals illustrating high $O_{2}\mathrm{Hb}$ (r=0.57) and low HHb (r=0.03) temporal correlations in the middle frontal cortex of channel 24 during the spatial WM task. Shown peripheral ear signal had already been shifted (+0.7  s and −4.3  s for $O_{2}\mathrm{Hb}$ and HHb signals, respectively) according to the corresponding maximum correlation ($r_{\text{max}}$). Denoising was performed resulting in the low correlation (r=−0.13) between $O_{2}\mathrm{Hb}$ denoised (red dotted-line, left y-axis scale) and peripheral ear signals. HHb denoising was not performed due to lower correlation than 0.3 of threshold. Shaded-gray area indicated the task period of spatial WM (target stimulus and delay intervals, 8.5 s in total).

**Fig. 7:**
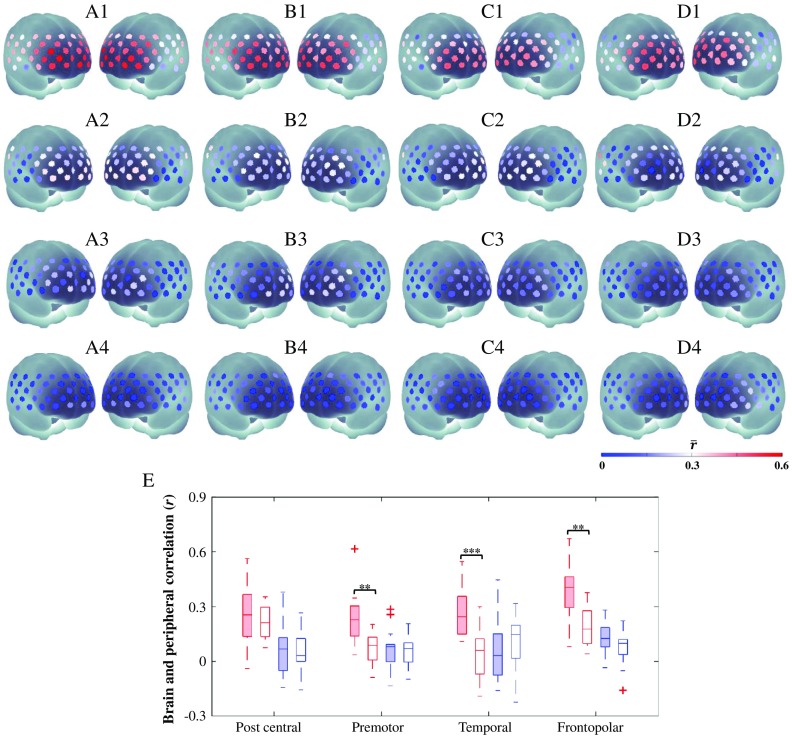
Subject-average $O_{2}\mathrm{Hb}$ (A1–D2) and HHb (A3–D4) correlation maps ($r_{\text{max}}$; inverse z-transform) of prefrontal and peripheral signals measured from ears (A1–D1, A3–D3) and finger (A2–D2, A4–D4) during spatial WM (A1–4), motor control (B1–4), eyes-open (C1–4), and eyes-closed RS (D1–4). $O_{2}\mathrm{Hb}$ correlation variances across subjects (E) showing significant differences of peripheral sources (red-filled and red-void boxplots for ear and finger signals, respectively) in premotor, temporal, and prefrontal cortices – p<0.001^(***)^, p<0.01^(**)^ for paired-sample t-test. There was no observed effect of peripheral sources in HHb correlations of all regions (blue-filled and blue-void boxplots for ear and finger signals, respectively).

Figure 6 shows the $O_{2}\mathrm{Hb}$ and HHb correlations between the fNIRS and shifted peripheral ear signals from a representative subject. The fNIRS signal came from channel 24 located in the right BA 45 in approximate. Because $r_{\text{max}}$ for the $O_{2}\mathrm{Hb}$ signal [i.e., 0.57; Fig. 6(a)] is greater than the threshold, $O_{2}\mathrm{Hb}$ denoising was done in that channel. $r_{\text{max}}$ for the HHb signal is low [i.e., 0.03; Fig. 6(b)]; thus, the HHb signal of channel 24 was left untouched (red-dotted line). Figure 7 presents the subject-average correlation (inverse z-transform) between channelwise fNIRS and peripheral PPG signals ($O_{2}\mathrm{Hb}$ and HHb) for all tasks. There was no significant task effect on both ($O_{2}\mathrm{Hb}$ and HHb) prefrontal-periphery correlation and optimum delay ($\tau_{\mathrm{opt}}$) using any peripheral sources. The $O_{2}\mathrm{Hb}$ $\tau_{\mathrm{opt}}$ was more positive (i.e., peripheral signal was ahead of the prefrontal signal, +0.8±1.5  s; mean±S.D.) than HHb $\tau_{\mathrm{opt}}$ (−0.6±2.2  s; mean±S.D.). $O_{2}\mathrm{Hb}$ signals measured in premotor, temporal, and frontopolar regions had the significantly higher correlation with peripheral signals measured in ears than in fingers [Fig. 7(e)]. Stronger correlation in the frontopolar was significantly observed compared to premotor (post-hoc Tukey–Kramer test; p<0.05) for $O_{2}\mathrm{Hb}$ prefrontal and peripheral ear signals. Meanwhile, peripheral finger signals showed strong relationship with $O_{2}\mathrm{Hb}$ signals of postcentral and frontopolar regions compared to premotor and temporal regions (post-hoc Tukey–Kramer test; p<0.05). The selection of peripheral source unlikely influenced HHb correlations [Figs. 7(A3)–7(D4)] and there was no observed spatial effect (interregions ANOVA, p>0.05). The lower correlation of HHb prefrontal and peripheral ear signals was significantly observed (CI>0.999) in all regions compared to the $O_{2}\mathrm{Hb}$ correlations [i.e., red-filled and blue-filled boxplots in Fig. 7(e)]. $O_{2}\mathrm{Hb}$ and HHb correlations between premotor-temporal and peripheral finger signals insignificantly differed. However, postcentral and frontopolar regions showed higher $O_{2}\mathrm{Hb}$ correlation compared to HHb correlation toward the peripheral finger signal [i.e., red-void and blue-void boxplots in Fig. 7(e)]. These suggested that (1) HHb signal might be less affected by peripheral shared systemic noise, (2) peripheral ear signals shared more similar components with prefrontal signals than peripheral finger signals, and (3) the effect of systemic noise on $O_{2}\mathrm{Hb}$ signal was inhomogeneous over the PFC.

### 3.3 Efficiency of Denoising

**Fig. 8:**
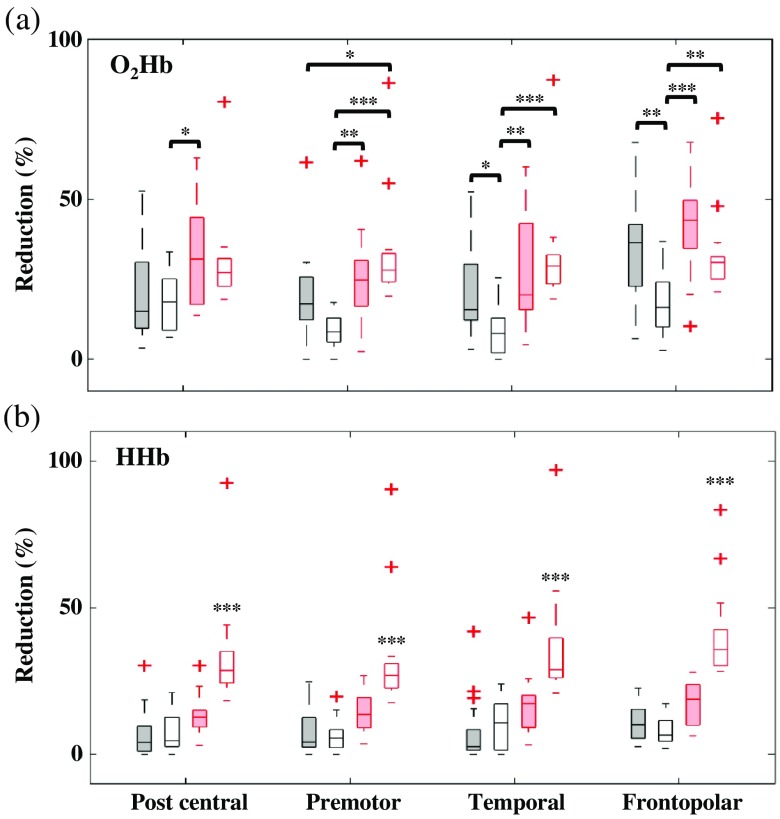
Subject variances of systemic noise reduction in (a) $O_{2}\mathrm{Hb}$ and (b) HHb signals neglecting the task effect in four channel regions: postcentral, premotor, temporal, and prefrontal cortices performed by ear-GLM denoising (black-filled boxplot), finger-GLM denoising (black-void boxplot), dual peripheral-GLM denoising (red-filled boxplot), and PCA 95 (red-void boxplot) – p<0.001^(***)^, p<0.01^(**)^, p<0.05^(*)^ for ANOVA and post-hoc Tukey–Kramer test. Because of low correlations between prefrontal and peripheral signals in HHb parameter, HHb signals were mostly not denoised. Therefore, the performance of peripheral-GLM denoising in HHb signal was significantly lower than PCA 95.

Denoising performance was compared in all signals (i.e., $O_{2}\mathrm{Hb}$ and HHb) and all tasks (i.e., spatial WM and other control tasks) using the current peripheral-GLM method (i.e., ear, finger, and both signals) and PCA 95. For signal reduction parameter, the task effect was initially evaluated. The signal reduction was comparable across tasks in all signal types and methods. Figure 8 shows subject variances in $O_{2}\mathrm{Hb}$ and HHb reductions using several denoising methods corresponding to four major regions. There was no spatial effect (ANOVA, p>0.05) on $O_{2}\mathrm{Hb}$ and HHb reductions using PCA 95. Performance of $O_{2}\mathrm{Hb}$ reduction using the ear-GLM denoising was spatially related where the highest reduction happened in the frontopolar (34±16%, task-average max. in left BA 10/11 by 45±23%; mean±S.D.). Peripheral-GLM denoising using ear signals had comparable $O_{2}\mathrm{Hb}$ noise reduction to PCA 95 in all regions except premotor (post-hoc Tukey–Kramer test). By using both ear and finger signals, the denoising performance insignificantly differed compared to PCA 95. The performance of peripheral finger regressor was worse compared to the dual ear-finger regressors in all regions. HHb prefrontal and any peripheral signals presented low correlation in all regions (i.e., no spatial effect); thus, HHb signals were likely left unprocessed. Therefore, denoising performance of HHb peripheral-GLM method was significantly lower than PCA 95.

**Fig. 9:**
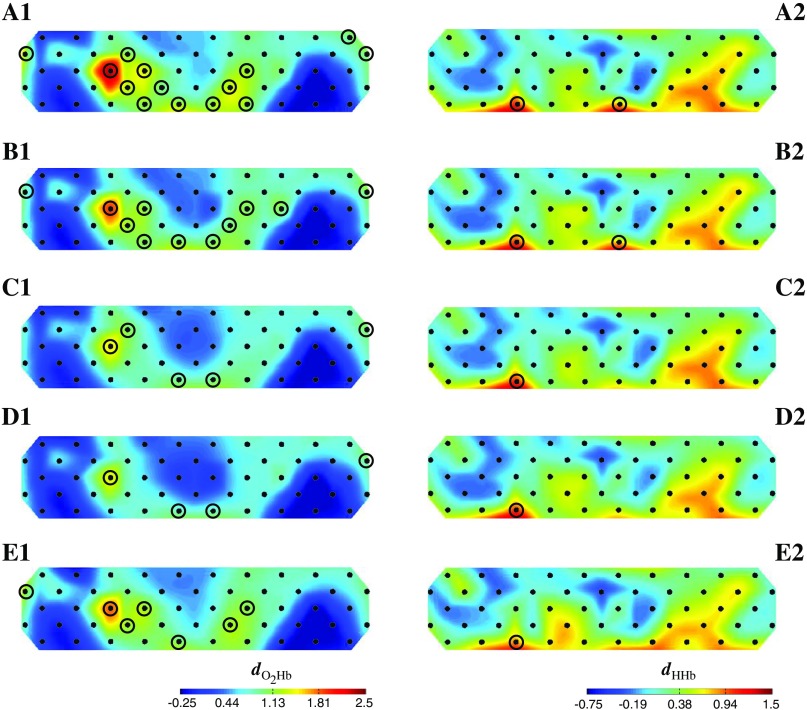
Cohen’s d effect size maps of $O_{2}\mathrm{Hb}$ (A1–E1) and HHb (A2–E2) activations for the spatial WM task before denoising (A1-2), after ear-GLM denoising (B1–2), finger-GLM denoising (C1–2), dual peripheral-GLM denoising (D1–2), and PCA 95 (E1–2). Black encircled channels indicate the significant activation channels (p<0.05; Holm–Bonferroni correction).

We consistently found no activation and no substantial effect size change of $O_{2}\mathrm{Hb}$ and HHb activations in control tasks after denoising using any methods. The denoising effect on activation changes was only observed in the spatial WM task. Figure 9 shows Cohen’s d effect size maps of $O_{2}\mathrm{Hb}$ and HHb activations during the spatial WM task after denoising. Denoising decreased the significances of $O_{2}\mathrm{Hb}$ increase (p<0.05; Holm–Bonferroni correction; black-circled channels) in group-analysis. The most decrease was performed by the dual-peripheral-GLM denoising [Fig. 9(D1)] showing more right lateralization (i.e., BA 45; channel 24). By using only ear signals as noise regressors, we still observed $O_{2}\mathrm{Hb}$ significance in the surrounding BA 45 compared to the finger-GLM and dual peripheral-GLM denoising. Regarding the low reduction of $O_{2}\mathrm{Hb}$ signal by finger-GLM denoising [Fig. 8(a)], the decreased significance of $O_{2}\mathrm{Hb}$ activation was unexpectedly observed. Furthermore, the change of HHb activation was not observed after denoising using any methods [Figs. 9(A2)–9(E2)]. These results were expected in the peripheral-GLM denoising due to low signal reduction; yet, PCA 95 presenting comparable signal reduction in both $O_{2}\mathrm{Hb}$ and HHb signals also showed the insignificant change of HHb activation. There was no significant difference of channelwise activation (i.e., $O_{2}\mathrm{Hb}$ and HHb) across methods.

## 4 Discussion

### 4.1 Direct Versus Multimodality Interpretations

Some physiological measurements, such as respiration rate by a pneumatic belt or arterial blood pressure by cuff,^45^^,^^65^ require additional interpretations to translate them into blood-related LF signals, before used as noise regressors in fNIRS studies. In our study, we directly measured the blood-related LF signals in peripheral sites using PPG, concurrently with the prefrontal fNIRS measurement. The peripheral LF signal is so-called “catch-all” signal that represents the combination of LF systemic physiological noises from various origins. It may include the fluctuations in heart rate (HR), arterial blood pressure (ABP), respiration (or downsampled version of them). Under the assumption that these signals affect both brain and peripherals based on vascular relations, the blood-related physiological signals can be used directly to denoise functional signals (i.e., fNIRS or fMRI) without knowing exactly the origins of these low-frequency oscillations (LFOs). The detailed benefits of such direct measurement can be found in the previous work of Tong et al. through simultaneous fMRI-NIRS measurement.^49^^,^^66^

### 4.2 Regressor Suitability

Cooper et al.^67^ previously used frontal and temporal lobes fNIRS signals as physiological regressors for fMRI data. However, an fNIRS signal with 3 cm of S-D distance is a mixture of both neuronal and nonneuronal signals from cortical and extracerebral layers, respectively. Directly using those as models could regress out the real neuronal signal. Concurrent finger measurements have been attempted to obtain the neuronal-free (i.e., systemic) signal as a noise regressor.^49^^,^^66^ However, noise regressor obtained from the ear has the following advantages: (1) the blood in ear is supplied by external carotid artery, which is close to the internal carotid artery (i.e., blood supply to brain); (2) the ear is easy to measure by our clip-equipped PPG probe with superior signal-to-noise ratio. As shown in Fig. 7, peripheral ear signals had the significantly higher correlation with prefrontal signals than those from fingers resulting in greater noise reduction (Fig. 8). This confirms our hypothesis that closer vascular distance may lead to high similarities between the signals. Despite the less signal reduction, the significance of $O_{2}\mathrm{Hb}$ activation in group analysis decreased after finger-GLM denoising. This suggested that denoising using less correlated signals could possibly regress out some signal portions. It was difficult to evaluate whether over-processing (i.e., spurious improved activation localization) actually happened or not. Therefore, the highly correlated noise model (e.g., peripheral ear signals) is more preferable for denoising.

In addition to sources of systemic noise regressor, we also investigated the regressor suitability between two signal types ($O_{2}\mathrm{Hb}$ versus HHb). The correlation between HHb prefrontal and peripheral (either ear or finger) signals is significantly lower than that of $O_{2}\mathrm{Hb}$ signals (Fig. 7). There are two arguments that may explain this HHb characteristic. First, HHb is less affected by the systemic noise. For example, according to Franceschini et al.,^18^ heart rate change significantly modulates the arterial compartment and is highly reflected in $O_{2}\mathrm{Hb}$; this is not the case for HHb in venous vasculature. Therefore, the HHb signal may be less prone to those systemic physiological fluctuations. This hypothesis is also consistent with the results reported by Sato et al.^68^ with the finding of lower correlation between HHb signal at brain and systemic skin blood flow (SBF) measured using laser Doppler flowmeter. Second, neither peripheral nor systemic SBF recordings are sufficient to model systemic noise in HHb signal. Even HHb extracerebral was found having low coherence with HHb brain.^69^ Previous studies reported that HHb brain is less sensitive and inconsistently responds toward cerebral blood flow change.^70^^,^^71^ In the current results, we found nonuniform HHb responses during the encoding interval across subjects resulting in the insignificance of HHb activation. The individual variances may implicate this issue. According to the above arguments, a global systemic variation in $O_{2}\mathrm{Hb}$ signal is a better noise regressor.

### 4.3 Systemic Denoising and Its Comparison to Other Denoising Methods

Katura et al.^72^ demonstrated the causal relationship between fNIRS signals and cardiovascular systemic noises modeled using HR and mean ABP during resting state. The study focused on a single channel signal around the posterior-superior temporal lobe and reported that the contribution of cardiovascular systemic noise up to 35% in $O_{2}\mathrm{Hb}$ and Hb-total signals. The remaining 65% portion was still unidentified noise components. In this study, we sought to isolate and remove systemic noise identified from peripheral sites. The reduction caused by systemic denoising (i.e., peripheral ear in $O_{2}\mathrm{Hb}$ signal), or also called as systemic noise contribution, was found to be around 25% to 34% in average and 39% to 58% in maximum (frontopolar channel 36; BA 10/11) across tasks.

From our results, we found that the systemic noise contribution was equal for any tasks. The spatial variability of systemic noise contribution was also observed over PFC as previously reported.^73^ Zhang et al.^74^ showed that LFO highly accumulated in $O_{2}\mathrm{Hb}$ signal over the frontopolar regions. This phenomenon may be explained by the fact that low signal sensitivity in the regions with big vessels (causing high absorption and low photon escape^75^^,^^76^). While the frontopolar is covered by the relatively small vessels (0.9 to 1.1 mm in diameter),^77^ the vessels in temporal regions are slightly greater than 2 mm.^78^ Apart from the above arguments, the task-evoked systemic noise might be colocalized in the draining scalp veins.^46^^,^^49^

In order to quantify the comparison between current and previous methods, we performed PCA 95 in the same datasets. PCA 95 is a well-known method to eliminate motion artifact with the assumption of less contribution of noise toward the signal. The arbitrary threshold of contribution cut-off (e.g., 95% and 80%) always become a limitation of PCA. Performance of PCA in signal reduction was uniform over PFC while the performance of peripheral-GLM denoising was spatially influenced. The $O_{2}\mathrm{Hb}$ signal reduction was comparable between (dual) peripheral-GLM denoising and PCA. After denoising, the significance of $O_{2}\mathrm{Hb}$ activation in group analysis was decreased by all the methods. There was no channelwise difference across methods in both $O_{2}\mathrm{Hb}$ and HHb activations despite the high reduction of HHb signal by PCA 95. This could suggest that (1) the current peripheral-GLM denoising performed comparably to PCA 95 in $O_{2}\mathrm{Hb}$ signals and (2) the HRF model for HHb signals might not be optimum. Uga et al.^79^ reported that the optimization of $\tau_{p}$ parameter could improve the observations of $O_{2}\mathrm{Hb}$ increase and HHb decrease. Even though the performance of peripheral-GLM denoising was equal to PCA, our current method could specifically remove systemic (i.e., nonneuronal) components rather than determining the assumption of signal contribution as PCA. Furthermore, PCA performance is influenced by the availability of channel number. Peripheral GLM denoising is potentially applicable for the required minimum measurement system.

Despite the potential results, we were still aware of the extracerebral (e.g., scalp) signal in the denoised signals. The scalp effect has been reported to be significant in PFC.^80^ One of the well-known methods to eliminate extracerebral signal is MD S-D separation.^36^ The characteristics of systemic denoising and MD S-D separation can be different from each other. External carotid artery supplying blood to ear shares the same upstream vasculature (i.e., common carotid artery) with internal carotid artery for brain supply. Any fluctuations in common carotid artery could affect downstream vessels in both brain and ears. Thus, the ear measurement is a reasonable interpretation of global systemic noise. In opposite, the MD S-D separation directly measures the regional changes in the superficial layer corresponding to nearby fNIRS channels. We believe that global systemic noise is also confined in the regional superficial signal due to similar blood vasculature as mentioned above. The impact of global systemic noise on regional superficial signal is still unknown. Furthermore, both systemic and extracerebral denoising have not yet been done together in fNIRS data analysis. We hope to address this issue in the future studies.

### 4.4 Task-Related Effect on Peripheral Ear Signals

There are two issues concerned with this subdiscussion. First, we have observed the high correlation between the $O_{2}\mathrm{Hb}$ frontopolar and peripheral ear signals for all tasks. The correlation distributions were also similar regardless of tasks (Fig. 7). Tachtsidis et al. and Sato et al.^68^^,^^81^ evidenced the high correlation of frontal lobe $O_{2}\mathrm{Hb}$ fNIRS with global changes of mean blood pressure and SBF, respectively. Kohno et al.^40^ also reported the effect of SBF contamination on the wide area of the forehead. This may implicate the high correlation between $O_{2}\mathrm{Hb}$ peripheral ear and SBF. This relationship is likely associated with the same vascular source of the common carotid artery.

**Fig. 10:**
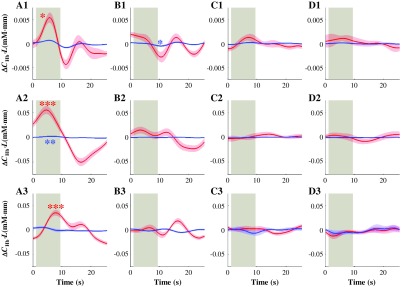
Subject-average ear (A1-D1), finger (A2–D2), and right BA 45 (channel 24, A3–D3) signals (red and blue lines for $O_{2}\mathrm{Hb}$ and HHb signals, respectively, with their standard errors) during (a) spatial WM, (b) motor control, (c) eyes-open, and (d) eyes-closed RS. The gray-shaded interval indicates the task period (target stimulus and delay intervals, 8.5 s in total). P<0.01^(***)^, p<0.05^(**)^, and p<0.1^(*)^ for one-sample t-test of $O_{2}\mathrm{Hb}$ increase and HHb decrease activations (β of encoding canonical HRF).

Second, we explored the task-related signals from peripheral measurements as shown in Figs. 10(A1)–10(D2). The averaged waveforms were different for all tasks. As performing the same task, ear and finger signals presented difference waveforms. Li et al.^82^ demonstrated the sensitivity difference on systemic manipulation responses (e.g., passive leg raising, paced breathing) in the ear, finger, and toe. We confirmed that there was no significant difference in $O_{2}\mathrm{Hb}$ and HHb activations between eyes-open and eyes-closed RS controls in both peripheral ear and finger signals. The significant task-related effect was observed in both prefrontal and peripheral (i.e., ear and finger) signals during the spatial WM task [Figs. 10(A1)–10(A3)]. Such effect was also reported in other tasks: verbal WM, finger tapping, visual checker-board, and anagram tasks.^36^^,^^68^^,^^81^ This finding is also consistent with the previous work of Kirilina et al.,^46^ where they found task-related activations in the superficial layer and concluded that “physiological origin of the systemic artifact is a task-evoked sympathetic arterial vasoconstriction followed by a decrease in venous volume.” These results comprehensively suggested that the tasks also simultaneously triggered the global systemic noises, which is consistent with the neurovascular coupling theorem arguing the local systemic regulation.^4^^,^^83^^,^^84^

Since the peripheral signals have task-evoked effects, there is a concern about the denoising method using the peripheral signals. We argue that the signal from the ears is a pure systemic physiological artifact. It does not contain any neuronal “contamination,” even it shows task-evoked changes. These changes are likely induced by global systemic changes as a result of performing tasks. Therefore, these peripheral signals are “noises” and have to be removed, regardless of their waveforms. Moreover, their waveforms (i.e., global systemic changes) should be different from the real brain activations, which is neuronal and regional [Figs. 10(A3)–10(D3)]. These global systemic changes are likely coupled and propagated into the blood-related change (i.e., flow, volume, and oxygenation) that affects the different brain and facial regions at the different time. Thus, in this study, in addition to accurately record the non-neuronal noise from ears, we adaptively remove it by temporally matching with each fNIRS channel before subtraction. As a result, we found the significant $O_{2}\mathrm{Hb}$ increase in DLPFC/VLPFC during the spatial WM task (Fig. 9) as previous studies^85^^–^^88^ even after denoising. This demonstrates that removing physiological noise with task-related waveform, we did not remove the task activation.

## 5 Conclusion

A high correlation between $O_{2}\mathrm{Hb}$ prefrontal and peripheral signals was observed consistently during all tasks, especially the peripheral signals from ears. Even without denoising process, $O_{2}\mathrm{Hb}$ activation was only detected in the spatial WM task. This suggested that the risks of false-positive activation in control tasks were rejected and fNIRS did measure the task-evoked activation. Although the task-evoked effect also globally appeared in the peripheral signals, the real neuronal task activation still remained after denoising. This denoising method had reduced the systemic noise up to 39% to 58% of raw signals. The denoising performance was comparable to PCA 95 and offered the advantages of specific systemic (i.e., nonneuronal) noise model, minimum measurement system (i.e., a peripheral channel versus multiprefrontal channels), and no required assumption of component contribution. Despite these promising results, the extracerebral noise that may regulate locally was not considered. Both global systemic and regional extracerebral noises should be managed to improve the analysis accuracy and this issue will be addressed in the future studies.
